# Validation of the Argentine version of the Montreal Cognitive Assessment Test (MOCA): A screening tool for Mild Cognitive Impairment and Mild Dementia in Elderly

**DOI:** 10.1590/1980-57642020dn14-020007

**Published:** 2020

**Authors:** Cecilia M. Serrano, Marcos Sorbara, Alexander Minond, John B. Finlay, Raul L. Arizaga, Monica Iturry, Patricia Martinez, Gabriela Heinemann, Celina Gagliardi, Andrea Serra, Florencia Ces Magliano, Darío Yacovino, María Martha Esnaola y Rojas, Adelaida Susana Ruiz, Héctor Gastón Graviotto

**Affiliations:** 1 Neurología Cognitiva y Neuropsicología. Hospital Dr. Cesar Milstein, Buenos Aires, Argentina.; 2 Carrera de Investigador Clínico del Gobierno de la Ciudad de Buenos Aires, Argentina.; 3 Carrera Interdisciplinaria de Especialización en Neuropsicología Clínica, Facultad de Psicologia, UBA, Buenos Aires, Argentina.; 4 Laboratorio de Memoria y Equilibrio, Buenos Aires, Argentina.; 5 Duke University School of Medicine, North Carolina, USA.

**Keywords:** mental status and dementia tests, cognitive dysfunction, Alzheimer’s disease, dementia, estado mental e testes de demência, disfunção cognitiva, doença de Alzheimer, demência, comprometimento cognitivo leve

## Abstract

The MoCA is a brief useful test to diagnose mild cognitive impairment (MCI) and mild dementia (MD). To date, no Argentine cross-cultural adapted validations of the Spanish version have been reported.

**Objective:**

To validate the MoCA in the elderly and study its usefulness in MCI and MD.

**Methods:**

This study included 399 individuals over 60 years old evaluated in the Cognitive-Behavioral Department (2017-2018). Patients with<3 years of education, sensory disturbances, psychiatric disorders, or moderate-severe dementia were excluded. The control group comprised cognitively normal subjects. Participants were classified according to neuropsychological assessment and clinical standard criteria into Control, MCI or MD groups. A locally adapted MoCA (MOCA-A) was administered to the patients and controls.

**Results:**

Mean educational level was 10.34 years (SD 3.5 years). MoCA-A score differed significantly among groups (p<0.0001). MoCA-A performance correlated with educational level (r: 0.406 p<0.00001). Adopting a cut-off score ≥25 (YI=0.55), the sensitivity for MCI was 84.8% and for MD ​​100%, with specificity of 69.7%. When adding a single point to the score in patients with ≤12 years of education, the specificity of the test reached 81%.

**Conclusion:**

The MoCA-A is an accurate reliable screening test for MCI and MD in Argentina.

## RESUMO.

O MoCA é um teste breve e útil para diagnosticar comprometimento cognitivo leve (CCL) e demência leve. Até o momento, nenhuma validação argentina com adaptação transcultural da versão em espanhol havia sido relatada.

**Objetivo:**

Validar o MoCA em idosos e estudar sua utilidade no CCL e demência leve.

**Métodos:**

Este estudo incluiu 399 indivíduos acima de 60 anos avaliados no departamento cognitivo-comportamental (2017-2018). Foram excluídos pacientes com menos de 3 anos de escolaridade, com distúrbios sensoriais, distúrbios psiquiátricos e demência moderada a grave. O grupo controle foi cognitivamente normal. Eles foram classificados de acordo com a avaliação neuropsicológica e os critérios clínicos padrão em Controles, MCI e demência leve. A versão adaptada do MoCA (MOCA-A) foi administrado aos pacientes e controles.

**Resultados:**

Média de escolaridade: 10,34 anos (DP: 3,5). O escore MoCA-A foi significativamente diferente entre os grupos (p<0,0001). O MoCA-A correlacionou-se com a escolaridade (r=0,406 p<0,00001). Com uma pontuação de corte ≥25 (IY=0,55), a sensibilidade para CCL foi de 84,8% e para demência leve 100%, com especificidade de 69,7%. Adicionando um ponto único à pontuação em pacientes com menos de 12 anos de escolaridade, a especificidade do teste atingiu 81%.

**Conclusão:**

O MoCA-A é um teste de rastreamento preciso e confiável para MCI e demência leve na Argentina.

The prevalence of dementia in Latin America and the Caribbean is 6-6.5/100 adults over 60 years old. From 2001 to 2040, a 77% increase in the number of people with dementia is expected in Argentina and Chile.^1^ There is a growing need for a brief reliable instrument for detecting dementia in its early stages that can be used in both daily clinical practice and treatment trials.^2^

The Mini-Mental State Examination (MMSE) has long been the most widely used screening test for cognitive impairment in the clinical setting and the research field.^1^

The Montreal Cognitive Assessment (MoCA) test was created as an instrument for the detection of Mild Cognitive Impairment (MCI) and Alzheimer’s disease (AD).^2^ This test was developed to overcome the limitations of the MMSE in diagnosis and differentiation between AD and MCI. The MoCA test requires approximately 10 to 15 minutes to administer. It includes 11 subtests evaluating aspects of attention, executive functions, memory, language, visuoconstructional skills, and orientation. Original instructions also observed that one point should be added to the total score in individuals with 12 or fewer years of education.^2^

In the original validation study, with a cut-off ≥26, the MoCA test achieved a screening sensitivity of 90% and 100% for MCI and mild AD respectively, with a specificity of 87%.^2^

The first Spanish version of the MoCA (MoCA-S), validated in Spain by Lozano et al.,^3^ was less effective than its original version for the screening of MCI.

In reference to Latin America and the Caribbean, the first publications reported date from 2013, i.e. ten years after creation of the test.^1^ Latin America is a region that includes a wide variety of heterogeneous nations and therefore, a standardized assessment instrument is required for each country. In addition, the analysis of the effect of education in developing countries is extremely important due to the high percentage of individuals with a low level of education.^1^

Regarding the use of cut-off points for the diagnosis of cognitive impairment, several studies have used the cut-off point of the original study.^2^ However, some authors have proposed alternative cut-off points, of 21^4^^-^ 6 or 23 points^7^ for MCI, and 14^4^ or 20 points^6^ for mild dementia. The study of Pereira-Manrique and Reyes^4^ included different cut-off points according to education level. The biggest drawback of the MoCA is its high educational bias, thus the original version recommends the addition of a point if educational level is less than 12 years.^2^ However, in populations with a low level of education, adding a single point may be insufficient.^8^

A recent systematic review demonstrated that a ≤25-point cutoff could lead to a high rate of false positive diagnoses of cognitive impairment; therefore, the authors suggested a cutoff of ≤22 points.^9^

A Brazilian study, including a wider range of educational levels, used the more conservative cut-off limit (≤22 points) and found that 67% of their control sample was regarded as cognitively impaired.^10^ In another recent Brazilian study, the most accurate MoCA cutoff was 15 points for dementia diagnosis, while the cutoff was 19 points for MCI diagnosis in a population with heterogeneous educational levels.^11^

Again, the heterogeneity in cut-off point estimations represents one of the main limitations to the use of the MoCA.

To date, only one valid MoCA test for screening MCI has been available in Argentina, although this version lacked cross-cultural adaptation to the local environment.^12^

According to various authors, the adaptation of the original version, to compensate for the educational and cultural bias in low and middle-income countries, implies not only a change of cut-off point, but also linguistic and cultural changes that allow a more reliable evaluation.^1^ An example of the importance of cross-cultural adaptation arises from the study conducted by Del Brutto et al.,^13^ in which more than 70% of the participants could not name ‘rinoceronte’ (Rhino) in the naming subtest, believing it was a “vaca” (Cow) , a more common animal in the Ecuadorian rural environment. Likewise in the version of Delgado et al.,^6^ the word “cara” (Face) was changed to “Rostro” (Face) and “Comuna” (Commune) to “localidad” (Town).

The MoCA-S is a screening instrument that could help to identify patients with cognitive impairment and optimize the use of public health resources in a country with limited economic resources.

The objectives of this study are to evaluate the psychometric properties and discriminative validity of the Argentine version of the MoCA (MoCA-A) in older adults, and to determine the optimal cut-off point of MoCA-A as screening tool for MCI and MD.

## Methods

### Design

A retrospective, cross sectional validation study of a test for the detection or screening of cognitive impairment-dementia was conducted.

### Description of MoCA-A Test

The MoCA is a one-page test with a total score of 30 points administered in approximately 10 minutes. The official Spanish MoCA-S version (http//www.mocatest.org/pdf files/test/MoCA-Test-Spanish.pdf) was adapted to our cultural environment (MOCA-A). The MoCA Clinic & Institute Quebec, Canada, were contacted to validate this local adaptation in 2018. Based on Argentine culture, taking the category and frequency of linguistic equivalents into consideration, the Expert Linguists Committee changed the words: “rostro” to “cara” (face), “seda” (silk) to “terciopelo” (velvet), and “clavel” (carnation) to “margarita” (daisy). “Rostro” is a very low frequency word in Argentina (when referring to “face” the word “cara” is used). We use “margarita” because carnation is easily associated with the color red (such as parrot and green; chicken and yellow, or sky and blue). Therefore, the fourth word facilitates recall of the fifth. This, however, does not happen with “margarita” (daisy) or “rojo” (red).

The test is scored according to the author’s recommendations, under 7 sub-items: *Visuospatial/executive* (short Trail Making Test B, copying a 3D cube, Clock drawing by command); *Naming* (naming of 3 animal figures); *Memory* (learning of a 4-word list with delayed recall); *Attention* (direct and inverse span, interference inhibition test and serial subtraction of 100-7), *Language* (repetition of 2 complex sentences and phonological fluency); *Abstraction* (quick analogies test) and *Temporospatial orientation* (www.mocatest.org).^2^

### Population

The study included individuals from urban area of Buenos Aires, over 60 years of age and evaluated in an ambulatory care setting between September 2017 and May 2018. Patients who were illiterate (<3 years of education), had significant sensory deficits, decompensated medical conditions, prior psychiatric disorders, previously diagnosed with moderate-to-severe dementia, and institutionalized subjects were excluded from the study. The diagnosis of dementia was made according to the Diagnostic and Statistical Manual of Mental Disorders 5^th^ edition (DSM-V)^14^ and NIA-AA^15^ criteria. For the diagnosis of MCI, the criteria of Petersen^16^ and Albert^17^ were used.

The control group were subjects without cognitive complaints and normal neuropsychological scores. The Control Group consisted of a series of asymptomatic healthy subjects that were part of a normative data group in our laboratory. Subjects were recruited from among clinic personnel, friends and family members of the authors as well as healthy relatives of patients.

### Clinical and neuropsychological assessment

Each participant in this study was evaluated by a neuropsychological and neuropsychiatric standard battery applied at the Cognitive Neurology Department of a Monovalent Hospital for Older Adults as part of the diagnostic assessment. Neuroimaging evaluated by an independent provider and blood analysis were obtained in order to exclude other secondary causes of cognitive impairment. The neuropsychological battery consisted of the following tests: the MMSE,^18^ Clock Test,^19^ Signoret Verbal Memory Battery,^20^ Trail Making Test^21^ (TMT A and B), direct and inverse digit span,^22^ Boston Naming Test - 60 item,^23^ semantic (animals) and phonological (words starting with P) verbal fluency,^24^ and the Clinical Dementia Rating Scale^25^ (CDR). The neuropsychiatric battery consisted of the Neuropsychiatric Inventory (NPI)^26^ and Beck’s Depression Inventory.^27^ The MoCA-A was administered to each subject, on the same day, and the neuropsychological evaluation was then performed.

Definitive diagnosis of the patients was based on a consensus between neurologists and neuropsychologists, according to the diagnostic test results, excluding the MoCA-A.

The MoCA-A was reapplied to subsamples of participants 10 days after the first assessment to calculate inter- and intra-rater reliability.

### Ethical considerations

This study was approved by the Ethics Committee of the Dr Cesar Milstein Hospital. Clinical work was subject to the Rules of Good Clinical Practice of the ICH, according to the last revision of the Helsinki declaration,^28^ and conformed with the principles of the 3301 Law of CABA (Autonomous City of Buenos Aires) on the protection of rights of subjects in health investigations.^29^

### Statistical analysis

Minimum sample size was estimated for the healthy Control, MCI and MD groups using likelihood ratio contingency tables, considering a unilateral hypothesis, prevalence and ratio of false positives, considering Fleiss’s correction and aiming for a minimum of 70% sensitivity and specificity.

To determine the discriminative validity of the MoCA-A, the following analysis was performed: age and education means were compared across the 3 subgroups using ANOVA with Bonferroni’s post-hoc correction. Receiver Operating Characteristics (ROC) curves were plotted, and the Area below the Curve (ABC) was compared between groups. Sensitivity, specificity and positive and negative predictive values were then calculated for each cut-off value of the test. The cut-off for maximum performance was calculated using the Youden Index, prioritizing sensitivity over specificity. The convergent validity between the MoCA-A and MMSE was determined through Pearson’s correlation analysis. To assess the internal consistencies of the MoCA-A, Cronbach’s alpha was calculated. Normative data was analyzed in the Control group (subjects with normal cognition). The Test-retest and inter-observer reliability data were evaluated using a two-tailed Spearman correlation index.

Statistical analysis was performed using version 25 of the SPSS statistics software (IBM Corp. Released 2017. IBM SPSS Statistics for Windows, Version 25.0. Armonk, NY: IBM Corp.).

## RESULTS

### Sample characteristics

Of the 399 subjects assessed, 155 were included in the healthy Control group, without cognitive complaints (CDR: 0), 158 in the mild cognitive impairment (MCI) group (CDR: 0.5), and 86 in the mild dementia (MD) group (CDR: 1). Demographic characteristics: 66.9% were women, and subject age ranged from 60 to 91 years, mean age 73.4 (SD 6.9) years. Regarding educational level of the sample, subjects had a mean of 10.34 years of schooling (SD 3.5). See Table 1.

**Table 1:** Demographic data.

Data	Demographics	Nº	%
Gender	Female	279	69.9
	Male	120	30.1
Age	60-69	130	32.6
	70-79	184	46.1
	≥80	85	21.3
Schooling level (years)	4-11	311	77.9
	≥12	88	22.1
Diagnostic	Control	155	38.8
	MCI	158	39.6
	MD	86	21.6

MCI: mild cognitive impairment; MD: mild dementia.

The Control group had a mean MoCA-A score of 25.46 (SD 2.26) and mean MMSE score of 28.66 (SD 1.44); the MCI group had a mean MoCA-A score of 20.60 (SD 3.5) and mean MMSE of 26.71 (SD 3, 5); while the MD group had a mean MoCA-A score of 12 (SD 3.8) and mean MMSE of 21.95 (SD 2.8).

No significant differences were found forage of the Control sample and MCI patients. However, significant differences were found between these two subgroups and patients with MD (p<0.0001).

Regarding educational level stratified by diagnosis, no significant differences were found between Control and MCI groups, but there were significant differences between these two subgroups and the MD group (p<0.0001). See Table 2.

**Table 2:** Demographic characteristics and MoCA performance by subgroups.

	Subgroup (n)			
	Control (n=155)	MCI (n=158)	Mild dementia (n=86)	p-value (ANOVA with Bonferroni analysis)
Age - mean years (SD)	71.47 (6.2)	72.6 (6.25)	78.33 (7.3)	p<0.001^†^
Women (%)	74.8	74.8	70.9	p=0.01^†^
Schooling - mean years (SD)	11.2 (3.4)	10.67 (3.2)	8.11 (3.23)	p<0.001^†^
MoCA-A total score - mean (SD)	25.46 (2.2)	20.60 (3.5)	12.00 (3.8)	p<0.001*

† Significant differences were only found in control group vs mild dementia and MCI vs mild dementia.

* MoCA-A mean performance was significantly different between all subgroups.

### Normative data: MoCA-A Test in Controls (subjects with normal cognition)

Total MoCA-A score was positively correlated with years of education (r: 0.406 p<0.00001). Age, however, had no significant correlation (r: 0.06; p=0.23). No significant gender differences were observed (p=0.27).

The mean and Z MoCA-A scores for each stratum by age and educational level are reported in Tables 3 and 4. A cutoff limit corresponding to a z‐score ≤−1 was adopted for screening that favors higher sensitivity, and the cutoff corresponding to a z‐ score ≤−1.5 was considered for screening that favors higher specificity. Individuals with MCI typically perform within the −1.0 to −2.0 range. Individuals with dementia typically perform with scores below −2.0.

**Table 3:** MoCA-A control scores stratified by age and schooling level.

Age	Schooling	Mean	N	Standard deviation
60 a 79 years	<12 years	24.72	47	2.12
	≥12 years	27.12	16	2.02
	Total	25.33	63	2.33
70 a 79 years	<12 years	25.13	53	2.16
	≥12 years	26.70	20	2.17
	Total	25.56	73	2.26
Older than 80 years	<12 years	24.81	11	2.04
	≥12 years	26.62	8	1.92
	Total	25.57	19	2.14

Normative data extracted from Controls (subjects with normal cognition); MoCA-A: Montreal Cognitive Assessment adapted to Argentine population.

**Table 4:** Cutoffs for the MoCA total score by age and education level.

Age	Z score	Years of schooling		
		≤7	8 to 12	>12
60-64	≤-1	22	23	25
	≤-1,5	21	21	24
	≤-2	20	21	24
65-69	≤-1	22	22	25
	≤-1,5	21	21	24
	≤-2	20	20	23
70-74	≤-1	21	23	25
	≤-1,5	20	22	24
	≤-2	19	21	23
75-79	≤-1	24	24	26
	≤-1,5	23	24	26
	≤-2	22	23	25
> 80	≤-1	22	23	25
	≤-1,5	21	22	24
	≤-2	20	21	23

Cutoffs for the MoCA-A total score not including an extra point for low education.

### Psychometric properties of the MoCA-A Test

#### Scale evaluation

Test-retest and inter-observer reliability data were collected from a subsample of 34 participants (patients and healthy subjects) and Spearman correlation indexes were calculated.

A Spearman correlation index of rs=0.818 (p<0.001) was obtained for inter-observer reliability and rs=0.949 (p<0.001) for test-retest reliability. These results suggest good stability over time and a strong correlation between different observers.

MoCA-A internal consistency was assessed using the Cronbach alpha index, which yielded a score of 0.8866 for the different items assessed. This value is similar to that obtained in the original scale validation.^2^

Finally, a significant convergent validity between MoCA-A and MMSE scores was found based on the Spearman correlation index (rs= 0.710, p<0.0001).

### Discriminative validity of MoCA-A

**Figure 1:**
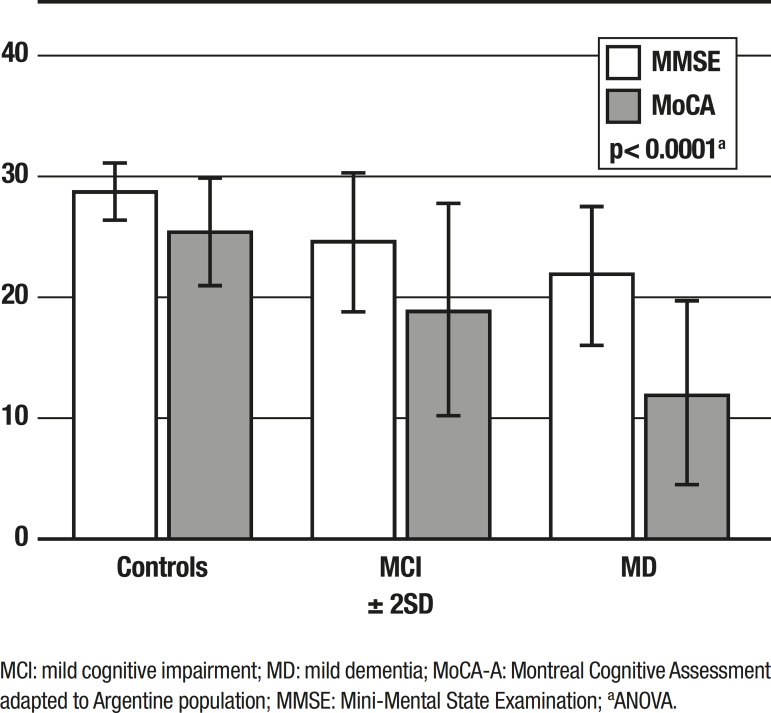
Performance in MMSE and MoCA-A by subgroups. MCI: mild cognitive impairment; MD: mild dementia; MoCA-A: Montreal Cognitive Assessment adapted to Argentine population; MMSE: Mini-Mental State Examination; ªANOVA.

The test was evaluated comparing the MoCA-A result with the diagnosis made by the neuropsychological battery, revealing a strong concordance between both measures (kappa of 0.69 95% CI 0.54-0.69). Furthermore, ANOVA was performed to correlate mean MoCA-A scores among the three subgroups. The MoCA-A score was differed significantly among all three subgroups (p<0.0001). See Figure 1.

#### ROC curve analysis

The ROC curves for the MoCA-A were plotted for MCI vs. Controls and for MD vs. Controls.

**Figure 2:**
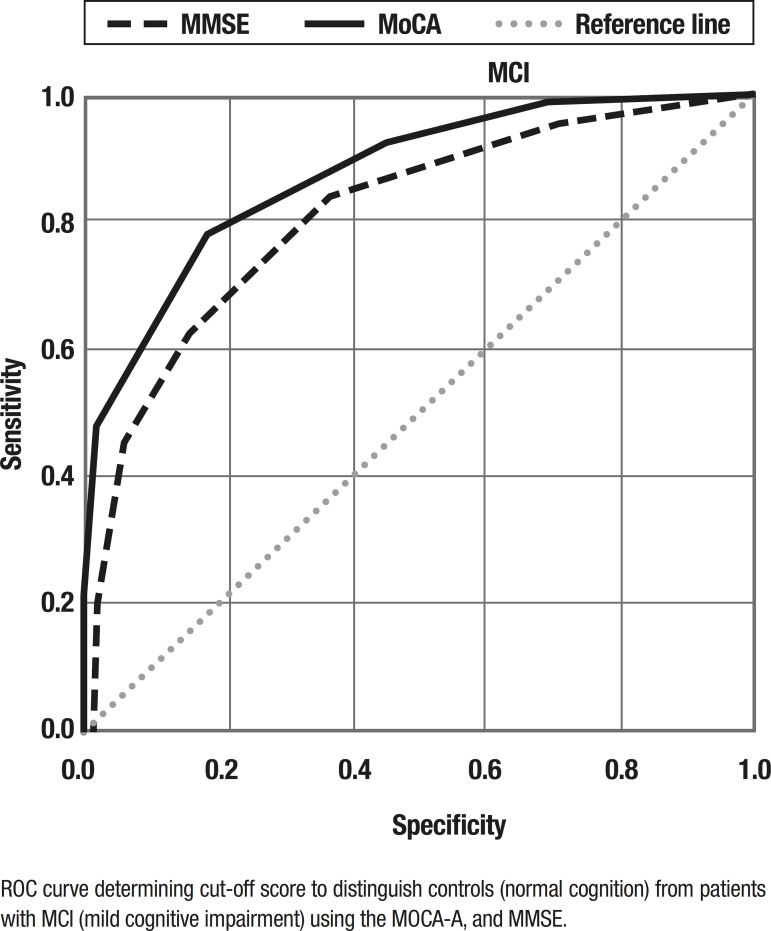
ROC curves for MMSE and MoCA tests for the detection of MCI. ROC curve determining cut-off score to distinguish controls (normal cognition) from patients with MCI (mild cognitive impairment) using the MOCA-A, and MMSE.

The ROC curves are presented in Figure 2 and the area under the ROC curve was 0.877 (95% CI [0.841-0.914]) for MCI and 0.99 (95% CI [0.99-1]) for MD.

The corresponding values for the MMSE were 0.812 (95% CI [0.764-0.860]) for MCI and 0.975 (95% CI [0.952-0.999] for MD.

#### Sensitivity and specificity

Sensitivity and specificity of the test were optimized for the population sample by using a cut-off point ≥25 (YI=0.55) (scores ≤24 were considered an abnormal result). With this cut-off point, a better balance between sensitivity and specificity was achieved. Sensitivity of the test to detect MCI was 84.8% and for MD was 100%. Specificity was defined as the percentage of controls that scored greater than or equal to the cutoff score of 25.

MoCA-A had a specificity of 69.7%. The positive predictive value was 74% for MCI and 65% for MD, while the negative predictive value was 82% and 100%, respectively.

### Effect of educational level

Educational level had some effect on test performance. The MoCA-A scores were lower for subjects with less education in all three subgroups. In subjects with ≤12 years of education, the MoCA-A (ABC=0.88) showed a better discrimination capacity than the MMSE (ABC=0.83). Adding a point at the end of the evaluation in patients with ≤12 years of education, in accordance with the original recommendation of the test designers,^2^ partially reduced the effect of educational level, increasing the specificity of the test to 81%.

## DISCUSSION

In this study, we presented the first cross-cultural adaptation of the MoCA test in an urban area of Buenos Aires, Argentina. The present study yielded a cut-off score ≥25 for screening MCI and MD in elderly, and the normative data of this Argentine local adapted version (MoCA-A).

One limitation of our study is that we excluded illiterate individuals. Gómez et al.,^32^ in a Colombian elderly population, reported that illiterate individuals and those with less than 5 years of education had a mean score of 17, whereas those who had completed primary school (5 years) or had more than 5 years of education scored 18 and 21, respectively. Therefore, we cannot extrapolate our results to the cited population.

Here we have demonstrated an accurate classification of the sample population in concordance with the results from the neuropsychological battery. In the present study, MoCA-A sensitivity using a cut-off of ≥25 was 85% for the detection of MCI and 100% for the detection of dementia in early stages. By contrast, sensitivity of the MMSE with a cut-off ≥26 was of 61% for the detection of MCI and 97% for mild dementia. A significant cognitive pattern arose for the performance on the MMSE and MoCA-A in the different subgroups (Figure 1): the majority of the control patients scored within the normal range, and the majority of the patients with mild dementia scored within the abnormal range for both tests. MCI patients, however, obtained abnormal scores on the MoCA-A, but normal scores on the MMSE.

The specificity of MoCA-A was 70%, which is lower than the specificity of the MMSE (86%). Nevertheless, due to the fact that the MoCA was designed as a screening test, it is intended to identify people with cognitive disorders (high sensitivity) and refer them for neuropsychological tests and full evaluations in memory clinics, reducing unnecessary health system costs.

Currently, there is no single screening tool that allows the primary care provider to quickly cover the wide range of cognitive impairment stages observed in the clinic. The MoCA-A has proven to be a useful test for mild stages of cognitive impairment (MCI and MD),^2^ while the MMSE might be superior for dementia follow-up in more advanced stages.^2^

Given the correlation between MoCA-A performance and educational level, subjects with low schooling achieved lower scores.^3^ Adding a single point at the end of the test can minimize this effect, although test results should be carefully examined in low-educated populations, as described in other MoCA validations.^3^^-^ 14 ^,^ 31 ^,^ 32

The previous Argentine validation^12^ established a limit of ≥26, just one point higher than our study. This phenomenon may occur because the sample of González Palau’s study^12^ had a higher level of education. It is noteworthy that the mean years of education in the present study was closer to the Buenos Aires demographic data^33^ for the population aged ≥65 years (10.3 in present study vs 11.1 years) than the figure in the study by González Palau et al.^12^ (13.6 years).

Cognitive tests should be harmonized for use in health centers in different countries.^1^ Cultures differ substantially, even those with the same language, making it essential to have a correct translation and adaptation of the test to the local characteristics.

Here we demonstrated a reliable adaptation of the MoCA for an Argentinian population, taking into account educational discrepancies and differences in vocabulary. This study can increase the standard of care for Argentine patients with MCI and dementia and also provide a model which physicians in other Spanish-speaking cultures can use to adapt the MoCA to their own standards and populations.
